# Integrating Mental Health and Development: A Case Study of the BasicNeeds Model in Nepal

**DOI:** 10.1371/journal.pmed.1001261

**Published:** 2012-07-10

**Authors:** Shoba Raja, Chris Underhill, Padam Shrestha, Uma Sunder, Saju Mannarath, Sarah Kippen Wood, Vikram Patel

**Affiliations:** 1 BasicNeeds Policy and Practice Directorate, Bangalore, India; 2 BasicNeeds UK, Leamington Spa, United Kingdom; 3 Livelihoods Education and Development Society (LEADS), Pokhara, Nepal; 4 BasicNeeds Policy and Practice Directorate, Oakland, California, United States of America; 5 Faculty of Epidemiology and Population Health, London School of Hygiene and Tropical Medicine, London, United Kingdom; 6 Sangath, Goa, India

## Abstract

As one article in a series on Global Mental Health Practice, Shoba Raja and colleagues provide a case study of BasicNeeds in Nepal, which emphases user empowerment, community development, health systems strengthening, and policy change to help socially disadvantaged individuals with mental health conditions.

**Summary Points**

- The BasicNeeds model of Mental Health and Development (MHD) emphasizes user empowerment, community development, strengthening of health systems, and policy influencing.
- The MHD model works in partnership with governments to provide the “great push” that is required to set up services where mental health and development has not been a priority.
- The model is comprised of five key components: capacity building, community mental health, livelihoods, research, and management.
- Involving affected individuals, their families, and communities in a program, as well as tapping into local resources, is essential to the success and sustainability of a program.
- Strategic engagement with government and other stakeholders is critical to demonstrating a project's capacity to influence mental health practice and scale up.

*This case study is part of the* PLoS Medicine *series on Global Mental Health Practice.*

## Mental Health and Development

People who live in conditions of social disadvantage are at greater risk of developing mental illness [1]. Access to treatment in low- and middle-income countries (LMICs) is limited and can be expensive [2]. Stigma makes it difficult to secure already limited employment and education opportunities [3]. While a mental health treatment gap has been widely acknowledged, less attention has been paid to addressing the poverty gap, which often accompanies mental illness [4]. The recent World Health Organization (WHO) report on mental health and development concluded that people with mental health conditions met all the criteria for vulnerability and merit targeting by development strategies and plans [5].

**Figure 1:**
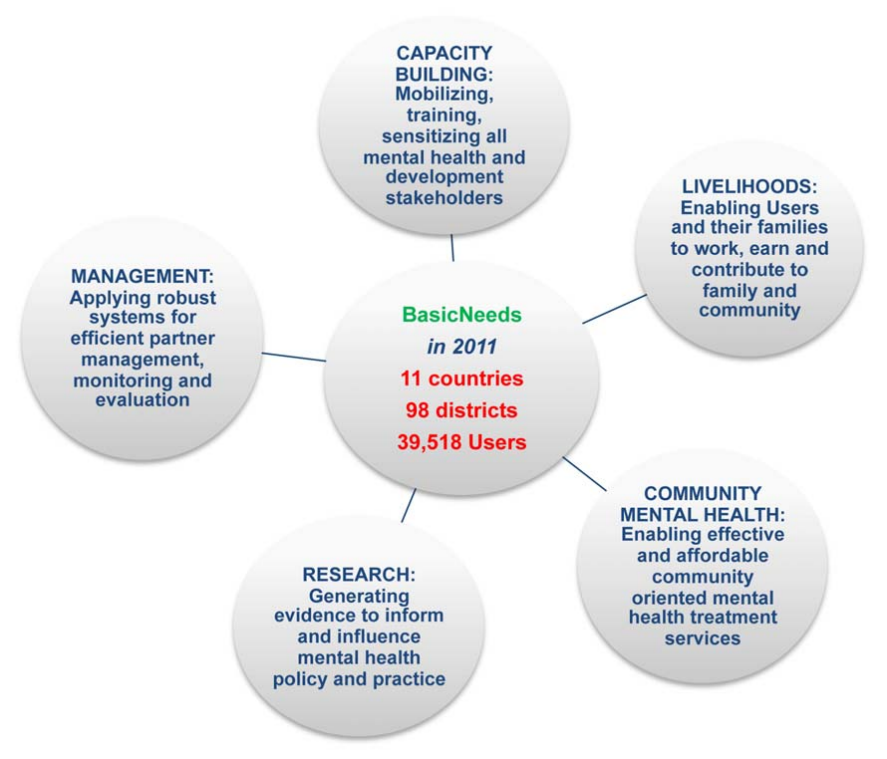
The BasicNeeds Mental Health and Development Model. The vision for the model is that the basic needs of all people with mental illness or epilepsy throughout the world are satisfied and their basic rights are recognized and respected. The purpose is to enable people with mental illness or epilepsy to live and work successfully in their communities.

BasicNeeds was founded in 2000 and developed its community-based integrated Mental Health and Development (MHD) model, inspired by development theory, which emphasizes user empowerment and community development, as well as strengthening health systems and influencing policy [6],[7]. Figure 1 shows each component of the MHD model.

In practice, the five modules of the MHD model work in conjunction to address the treatment, capabilities, and opportunities gaps experienced by affected individuals. Evidence suggests that community-based models that integrate health care and social interventions can have a positive impact on clinical outcomes and social and economic functioning for affected individuals in low-resource settings [8],[9]; and the BasicNeeds Model offers a feasible method of integrating mental health into existing community-based interventions [10].

BasicNeeds has witnessed exponential growth in response to requests for MHD programmes. In 2011, BasicNeeds operated MHD programmes in a total of 98 districts in 11 countries (Ghana, Uganda, Kenya, Tanzania, India, Sri Lanka, Nepal, Lao PDR, and Vietnam, with new programmes being initiated in China and the United Kingdom), working with 55 local partners, reaching 39,518 affected individuals. A major challenge has been sustaining existing programmes while adding new ones. After extensive consultations, BasicNeeds planned further scale up through a social franchise of the MHD model, i.e., a commercial franchising approach to replicate and share organizational models for greater social impact [11].

This paper will focus on a description of one particular MHD program in Nepal. The Nepal program was chosen because this allows highlighting operations in a fragile state where the government is unable to deliver even the most basic services, particularly in remote regions [12]. Nepal is also the first country where BasicNeeds has not set up a country office but operates through a direct partnership with an independent local nongovernmental organization, with expertise in community-based rehabilitation (CBR) and related training, called Livelihoods Education and Development Society (LEADS)—an operational prototype for future franchisees.

## MHD in Nepal—A Case Study

Nepal is a Himalayan country, sandwiched between China and India, with a population of 28.2 million people [13]. The country is divided into 75 districts and almost 90% of the population lives in rural areas [14]. Nepal's gross national income per capita at purchasing power parity (PPP) in 2010 was US$1210, ranking 148 out of 167 [15]. The life expectancy at birth is 68 years and the literacy rate is 59% [12]. Long-standing political conflict has created additional hardships. Less than 1% of health expenditure is spent on mental health (0.14%), and there is no mental health legislation [16]. Nepal currently has only one public sector psychiatric hospital offering inpatient services and 32 psychiatrists. United Mission Nepal's Community Mental Health programme between 1990 and 2004, despite challenges of sustainability, was an excellent effort in advocating for integrating mental health into primary care in Nepal [17],[18].

The Nepal MHD programme, funded through the Department for International Development, UK, is a 4-year programme (May 2010–March 2014) operating in Baglung and Myagdi districts, with populations of 270,009 and 113,731, respectively. The majority depend on agriculture for their livelihood. A baseline situational analysis revealed the absence of any government mental health services and an absence of mental health trained human resources. The economic burden for those who sought treatment was heavy [19], estimated at 25,000 Nepalese rupees (US$312) for a family per year [20].

**Figure 2:**
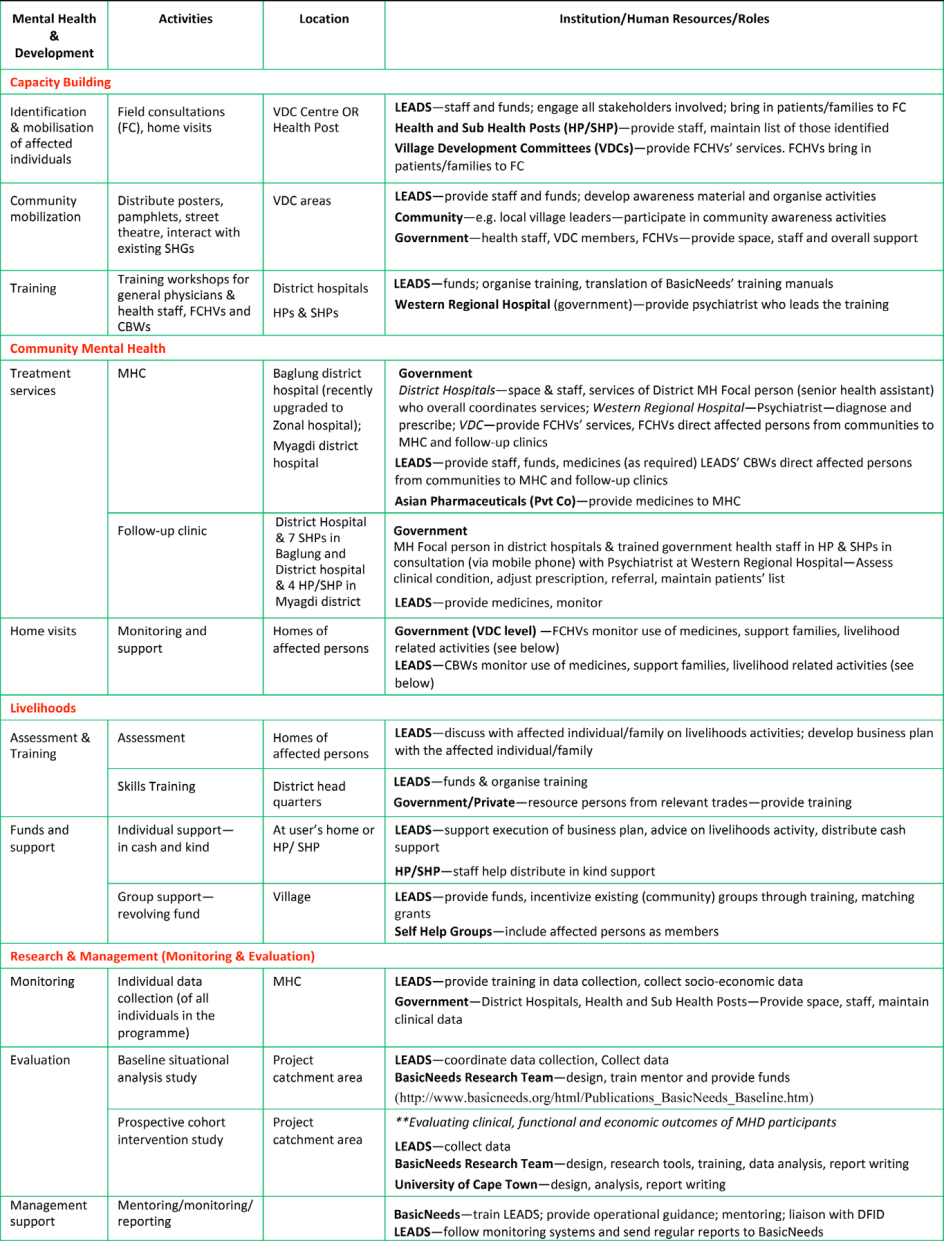
The Nepal Mental Health and Development programme matrix. Detailed description of key activities, locations, and resources pertaining to the Nepal Mental Health and Development Programme. *Mental Health Camp* is a concept popular in India, and refers to a collaborative activity in which a team of health professionals carry out out-patient clinics in community settings at regular intervals. *VDC (Village Development Committee)* is an elected government body at the lowest level of governance (small group of villages) in Nepal. *Primary Health Care* in Nepal is provided through a decentralized system. *Health Posts* (HP) cover an area of 3 to 4 *Sub Health Posts* (SHP). A *Health Assistant* (HA) is the In-Charge of a HP. SHP are established in all VDC areas. *Auxiliary Health Worker* (AHW) heads a SHP. Other staff in a SHP are Auxiliary Nurse Midwife, Maternal & Child Health Worker, and Village Health Worker. *Female Community Health Volunteers* (FCHVs) are volunteers attached to the SHP/HP and are involved in health education in their communities. They receive an annual incentive from the government. *Mothers' Groups* are community-level women's groups that are encouraged by the government through the VDCs and specifically linked to the primary health care facilities. Mothers' group meetings are facilitated by the FCHVs. *Community-Based Workers* (CBWs) are community-based staff recruited by LEADS for the project.

Figure 2 describes the programme matrix of the Nepal MHD model. The matrix demonstrates the role of diverse sectors in implementing the model, including the close links with the districts' government-run health facilities and existing community structures—a key strategy to integrate, and sustain, mental health and development.

**Figure 3:**
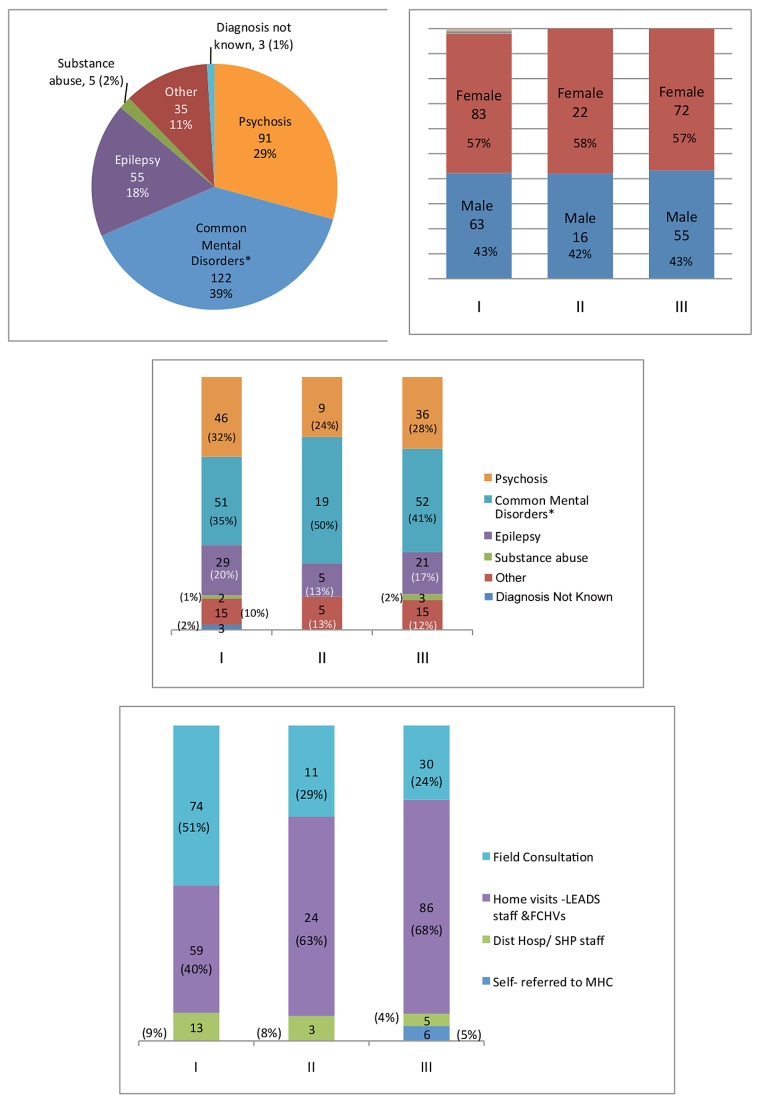
Characteristics and benefits of the users of the Nepal Mental Health and Development Programme. Upper left: Breakdown of diagnoses for all users of the program. Total number of users is 311, with 134 males and 177 females. Upper right: breakdown by gender of new individuals accessing treatment during the periods (I) July–September 2010, (II) October–December 2010, and (III) January–March 2011. Center: breakdown of diagnoses patterns for new individuals accessing treatment during each of the periods (I) July–September 2010, (II) October–December 2010, and (III) January–March 2011. Bottom: source of identification of new individuals accessing treatment during each of the periods (I) July–September 2010, (II) October–December 2010, and (III) January–March 2011. *Common mental disorders refers to anxiety, depression, phobia, and psychosomatic disorders.

The initial *identification* of affected individuals was done by appropriately trained key local stakeholders who mobilized these individuals to seek care from the mental health program (Figure 3 has details; [21]). Service provision followed a collaborative care model [22].

*Treatment services* started in August 2010 when the first Mental Health Camps (MHC) were held at the district hospitals in Baglung and Myagdi. Dr. Lumeshor, chief psychiatrist at the Western regional hospital (WRH), Pokhara, attends the camps with his team. The appointment of a senior health assistant as “mental health focal person” in November 2010 in both district hospitals greatly helped to manage the “flow” of mental health activities. However, it soon became clear that the district hospitals could not remain the only point of service provision. The number of patients increased but the frequency of the camps could not be increased, as the psychiatric team was unable to come more often. Besides, for many patients, accessing the hospitals meant four hours to walk each way. Thus, *follow-up* clinics were started at the Health Posts with the District Health Offices permitting the newly trained health personnel to run them. They, however, needed further coaching and supervision. LEADS provided them with SIM cards for their mobile phones, which they use on clinic days to maintain contact with the chief psychiatrist at WRH.

Starting in October 2010, individuals/families were prioritized for *livelihoods* support (diagnoses, process, and criteria for prioritizing, see next section) through skills training and/or cash grants for setting up a business or in kind. Simultaneously affected persons were linked into existing *self-help groups* (SHGs), opening up opportunities to integrate into mainstream groups and ensuing opportunities. LEADS' community-based workers (CBWs), coordinators, and female community health volunteers (FCHVs) made *home visits* to provide continuing support to the families and to also identify more affected individuals.

## Impact, Barriers, and Opportunities

Figure 3 provides an overview of the characteristics of and benefits for persons affected by mental illness accessing the MHD program in the short span of the 8 months since its inception.

The most common diagnoses were common mental disorders, followed by psychosis and epilepsy [23]. Qualified psychiatrists made diagnosis using WHO ICD-10 criteria, and thereafter recorded follow-up assessments in individual clinical information sheets. Of the 311 patients registered with the program until March 2011, 269 have been reported to show improvement. Over time we saw an increasing number of identifications from home visits and some self-referrals.

Baseline data collected at MHC showed 142 had accessed pharmacological treatment earlier, the vast majority from private providers in Kathmandu (4 days travel) or Pokhara (2 days). Apart from the travel costs, these families also paid for the consultation and medicines. All of them now attend MHC at the district hospital (4 hours travel maximum) and follow-up clinics in their local health posts, do not pay for services or medicines, are registered as Out Patient Department (OPD) patients, and are therefore part of the district health management information system (HMIS).

Of the 311 persons who have so far accessed the program, 32**/**214 (15%) of those who were not in an income-generating occupation began earning an income, and 22**/**48 (46**%**) of persons who were not engaged in any form of productive work (e.g., household chores) began such work. While this is low proportion relative to the estimated epidemiological need, the capacity of the health facilities requires further strengthening to provide mental health services to a larger number of patients.

Between October 2010 and March 2011, 55 affected individuals, showing significant clinical improvement, were assessed by LEADS for eligibility for livelihoods interventions. A checklist was used followed by discussions with the individuals themselves and their families. The indicators were: work before illness, interest to work, ability to work, traditional skills, family involvement, and market scope. Thirty-one individuals, with varying diagnoses (psychotic disorders-11, epilepsy-11, common mental disorders-9) were prioritized for support. In October 2011, LEADS carried out an evaluation of the outcomes of these 31 individuals. Data collected were: details of business plans, investment made, expenses incurred, income and savings details as well as their views about progress, problems, family support, financial situation, and future plans. Initial findings showed that all 31 were earning in a range of occupations including running a tea/grocery shop, chicken and goat rearing, tailoring, and embroidery. The six who earned prior to the program observed an increase in income ranging between 17% and 108%. Two individuals with epilepsy were doing skilled work (tailoring and making copper pots) and reported monthly earnings well above the stipulated minimum wage. Two persons diagnosed with depression, whose occupations were running a provision shop or tailoring, earned close to the minimum wage. The rest have incomes below the minimum wage. Ten have deposited savings with LEADS to be transferred into the account of a livelihoods co-operative that has been initiated.

The program has experienced a number of barriers in its implementation. Villages in both districts are remote, almost entirely inaccessible by road, and distances are still measured in number of days to walk. Despite the inhospitable terrain and associated difficulties, demand for services is growing and a key challenge is to keep pace with supply—i.e., availability of psychiatrists, trained health personnel, and medicines. At present, MHC held at the district hospitals every alternate month are the nearest point where/when the psychiatrist is available. LEADS is currently talking to a local private hospital for additional psychiatric support.

The increased or regained capacity of affected persons to work and earn has been a motivator. However, opportunities are few and earnings are low by any standards. The lack of development in the region limits the scope of available livelihoods options. The hilly terrain and sparse population makes it difficult to bring together a reasonable number of persons from different villages to form SHGs that can be sustained over time for self-advocacy. Integrating affected persons into the innumerable existing village-level SHGs (which can also help address stigma) posed problems, as existing members resisted the idea of mentally ill people joining. Incentivizing the SHGs with revolving micro-credit funds and skills training has helped to integrate affected persons to some extent.

In Nepal, primary health care is offered through a decentralized system [24]. The MHD programme already works through this. Continued engagement with health facilities, support to affected persons and families for livelihoods, and repeated awareness activities over time will help integrate the model into the routine activities of the existing providers and communities, but funds for sustaining these activities will be required. Continued political instability in Nepal has delayed LEADS' plans for engaging with the government more substantially.

## Looking to the Future

In the two districts the plan is to expand access and sustain the program by building capacity in local resources by training more local doctors in mental health (both private and government); holding MHC in remote locations so persons living there have easier access to specialist attention; training and supporting all health posts to include mental health records in HMIS; widening the scope of training for health workers and FCHVs to support livelihoods interventions; establishing a livelihoods cooperative; training affected persons to evaluate services; and forming district-level advocacy groups of affected persons. LEADS will step up its engagement with the Primary Health Care Revitalization Division for policy changes, especially on psychotropic medicines allowed at the primary care level and budgetary allocations for mental health. Ultimately, lessons from the experiences in Baglung and Myagdi, and evidence from a cohort intervention study (underway), will be used for designing a scaled-up programme in six more districts in the Western Region.

BasicNeeds has implemented the MHD model in nine countries. Many of the older programmes have encountered and negotiated the kind of difficulties we are currently observing in Nepal, and lessons from those experiences may have relevance in Nepal. In Uganda, for example, advocacy groups now engage directly with district officials to lobby for improved treatment services. In Ghana, groups have come together as a registered national association, the Mental Health Society of Ghana (MEHSOG), for advocacy. In Lao PDR, mental health services are available through primary care in nine districts of Vientiane capital region. There are a number of lessons from BasicNeeds' total experience in 10 years that can be relevant more widely in scaling up community-oriented mental health interventions in LMICs as well as developed countries.

Strategic engagement and effective working relationships with and involvement of government and other local/national stakeholders is critically important if a demonstration project has to influence mental health practice and policy for scale up. Involvement of affected persons and families is fundamental for maintaining relevance and effectiveness of interventions even if they are evidence based. Advocacy by affected persons is powerful and must be supported to become effective. Community involvement is important, as it supports affected persons and families in the process of recovery and can effectively support delivery of services. Involving affected persons, families, and communities requires detailed planning and has to be intrinsic to the intervention programme. Tapping into local or in-country resources, skills, and capabilities will help sustain service delivery. Designing simple yet rigorous records and data collection systems for complex community-based mental health programmes is feasible and crucial for monitoring quality and can substantially aid evaluations; such evaluations must be intrinsic to the intervention programme.

Above all, the MHD model is not in parallel or an alternative to government and other local efforts for effective mental health interventions. The model works to provide the “great push” required to set up mental health and development services in places where they are not on the agenda of government or civil society [25].

## Abbreviations

CBW: community-based worker
FCHV: female community health volunteer
HMIS: health management information system
LEADS: Livelihoods Education and Development Society
LMIC: low- and middle-income country
MHC: Mental Health Camps
MHD: Mental Health and Development
PPP: purchasing power parity
SHG: self-help group
WHO: World Health Organization
WRH: Western regional hospital
